# Evaluation of Internal and Superficial Self-Healing of Cracks in Concrete with Crystalline Admixtures

**DOI:** 10.3390/ma13214947

**Published:** 2020-11-04

**Authors:** Fabiana Ziegler, Angela Borges Masuero, Daniel Tregnago Pagnussat, Denise Carpena Coitinho Dal Molin

**Affiliations:** Post-Graduation in Civil Engineering, Construction and Infrastructure (PPGCI), Federal University of Rio Grande do Sul (UFRGS), Porto Alegre 90035-190, Brazil; angela.masuero@ufrgs.br (A.B.M.); daniel.pagnussat@ufrgs.br (D.T.P.); dmolin@ufrgs.br (D.C.C.D.M.)

**Keywords:** self-healing concrete, cracks, crystalline admixtures, chloride

## Abstract

Reinforced concrete structures are prone to cracking. The development of cementitious matrices with the capacity for self-healing soon after these cracks appear represents savings with inspections and repairs of the structures. Self-healing can be stimulated with the use of crystalline admixtures. Such materials easily react with water and increase the density of C-S-H (hydrated-calcium-silicate), forming insoluble deposits blocking existing pores and cracks. In this research, self-healing in concrete cracks was evaluated using three different crystalline admixtures, submitted to two and six wetting–drying cycles. The efficiency of self-healing was evaluated by optical microscopy and using the chloride diffusion test, which allowed calculating the predicted useful life of the concretes. The results highlight two important findings: (i) in optical microscopy, crystalline admixtures were not efficient in promoting self-healing on the surface of cracks in any of the studied concretes; (ii) the passage of chlorides by diffusion was lower for concretes with crystalline admixtures compared to the reference, showing better internal healing of these materials and, consequently, greater prediction of the concrete’s useful life.

## 1. Introduction

Concrete is a material widely used around the world in various construction techniques. Its purpose is to resist the efforts of compressive forces and protect the reinforcement from aggressive environmental agents, such as chlorides. Although it has been used for many years and has undergone several improvements, this cementitious material is susceptible to different sources of degradation, which can considerably compromise the durability of structures [1]. Among these degradations, cracks are found most frequently [2].

When exposed to the environment, reinforced concrete structures require periodic inspections to observe and monitor the presence of cracks and their geometries. Depending on the conditions and the location in which the structure is located, carrying out inspections may be difficult or impractical, in addition to the fact that such inspections and possible repairs require a high investment [3,4,5]. To avoid the deterioration of structures due to the combination of cracks and the action of chlorides, new techniques are necessary to ensure the repair of this manifestation of degradation soon after its appearance.

In this sense, the self-healing of cementitious materials has been the focus of several studies [6,7,8]. This phenomenon has water as its main activation mechanism, and it occurs from the continuous hydration of the cementitious matrix, especially in the early ages, while, in more advanced ages, the formation of CaCO_3_ occurs with the crack [9].

Autogenous self-healing in concretes can be stimulated using commercial products, such as crystalline admixtures, inserted in the cementitious matrix during its production [10,11]. Crystalline admixtures are classified as hydrophilic, which react easily when in contact with water, providing an increase in the density of C-S-H, forming insoluble deposits blocking existing pores and cracks, which support pressures of up to 14 bar [12].

The chemical composition of crystalline admixtures is formed by Portland cement, filler, pozzolans, sand, slag, siliceous powder, and “active chemicals” [2,13,14]. Diversified materials called crystalline admixtures are commercialized in the civil construction market, and their chemical compositions are kept confidential by the manufacturers.

The reaction of crystalline admixtures with cementitious compounds occurs according to Equation (1). The crystalline promoter (M*x*R*x*) reacts with C3S and water, producing modified C-S-H and a precipitated pore blocker (M*x*CaR*x*) in existing microcracks and capillaries [15,16,17].

3CaO-SiO_2_ + MXRX + H_2_O→CaXSiXOXR-(H_2_O)X + MXCaRX-(H_2_O)X. (1)

This can be written as calcium silicate + crystalline admixture + water → modified C-S-H + precipitated pore blocker.

The hydration reactions of crystalline admixtures spread throughout the concrete’s porous matrix, resulting in a system impermeable to water and other aggressive substances from the external environment and forming precipitated pore blockers [18].

Crystalline admixtures have advantages compared to other self-healing materials. For instance, they do not have to be encapsulated before being inserted into the concrete mixture [14]. Breugel [19] pointed out that, despite an initially higher cost of concrete with crystalline admixtures, the absence of repairs to structures with these materials can result in lower investments over time when compared to traditional concrete structures. However, there is a lack of research related to the physical and mechanical effects of using crystalline admixtures in cementitious matrices to convey confidence in the use of these materials in practice [16,20].

The performance of crystalline admixtures in the self-healing of cracks can be influenced by factors such as the thickness of the cracks and the exposure environment of the cement matrix. The control of the opening of a crack in concrete is one of the parameters for the efficiency of self-healing. De Belie et al. [10] mentioned that a cementitious matrix’s self-healing ability, without potentiating additions such as pozzolans or crystalline admixtures, can promote the closing of cracks of less than 0.3 mm, in the presence of water. The manufacturers of crystalline admixtures, in turn, point out that the use of such materials reinforces the ability of the cementitious matrix to seal cracks up to 0.4 mm.

According to Rooij et al. [21], the internal crack’s morphology may be responsible for the greater efficiency of self-healing when initiating this phenomenon in internal areas that present lesser thickness of the crack. This phenomenon can occur even in cases where greater thicknesses are verified on the sample surface, making it difficult to create a consensus on the maximum width capable of being healed. Thus, it is necessary to use test methodologies that make it possible to evaluate the internal self-healing behavior of cracks, such as the passage of chlorides through the diffusion test.

Different approaches and methodologies are used to assess self-healing, such as tests for the recovery of mechanical properties, the evaluation of the permeability of cementitious matrices, and the visual analysis of crack closure using microscopic images [22]. However, few studies use durability tests, especially the nonaccelerated chloride diffusion test, due to the greater demand for time when performing it to simulate real situations of concrete exposure to the passage of chlorides.

The environment that concretes are exposed to also influences the self-healing performance. The presence of water in the crack region is essential, as it activates the cement’s physical–chemical reaction and promotes the leaching of calcium hydroxide from the interior of the crack to the surface, intensifying the formation of calcium carbonate [23]. Cuenca et al. [24] concluded that the immersion condition of concrete samples with crystalline admixtures enabled a greater sealing of cracks with a thickness of up to 0.3 mm than the condition of exposure to air.

Roig-Flores et al. [7] analyzed concretes with crystalline admixtures in four exposure conditions: immersion, contact of one of the faces of the samples with water, chamber with 95% relative humidity (RH), and exposure to air at 40% RH. The authors defined the following order of efficiency for the closure of cracks, related to the environment of the samples: immersion in water > contact with water > wet curing > exposure to air.

On the other hand, Sisomphon, Copuroglu, and Koenders [25], when examining the recovery of a concrete sample’s mechanical properties containing crystalline admixtures when subjected to different exposure environments, concluded that cycles with 12 h of wetting and 12 h of drying were more favorable.

Reddy and Ravitheja [26] evaluated the ability to heal cracks between 0.2 and 0.4 mm in concretes with crystalline admixtures. The samples were exposed to immersion conditions, wetting and drying cycles, a sample with a surface in contact with water, and exposure to the natural environment. Through optical microscopy, the authors observed the complete surface sealing of the cracks in samples immersed in and subjected to wetting and drying cycles.

Thus, this research investigates the influence of three different crystalline admixtures on the ability to heal cracks in concrete. The cylindrical samples were cracked at 3 days of curing and subsequently subjected to two cycles (28 days) and six cycles (84 days) of wetting and drying to intensify the self-healing ability. The crack thickness measurements were performed at 3 days and at the end of each wetting and drying cycle, using an optical microscope. After the wetting and drying cycles, the cracked samples were subjected to the natural chloride diffusion test, with no potential difference applied, to simulate a real condition of exposure to the concretes. Through the chloride diffusion test, it was possible to forecast the service life of cracked concretes. The work made it possible to conclude the self-healing ability of cracks in concrete with crystalline admixtures and to compare the effects of these materials with the reference concrete.

## 2. Materials and Methods

### 2.1. Materials

#### 2.1.1. Cement

The cement used in this research was Portland cement composed with filler (CP II-F 40) (Votorantim, Rio Branco do Sul, Paraná, Brazil), according to NBR 11578 [27], equivalent to Type II cement by C150/C150M [28]. This cement was used because it is free of pozzolanic materials that could change the results with crystalline admixtures. The physical–chemical characteristics of cement are shown in Table 1.

#### 2.1.2. Aggregates

The aggregates were characterized according to sieve analysis by NBR NM 248 [29], while NBR NM 52 [30] was used for determining the mass specific gravity for fine aggregate and the NBR NM 53 [31] was used for coarse aggregate. The fineness module and the maximum dimension were determined according to NBR 7211 [32].

##### Fine Aggregate

**Figure 1:**
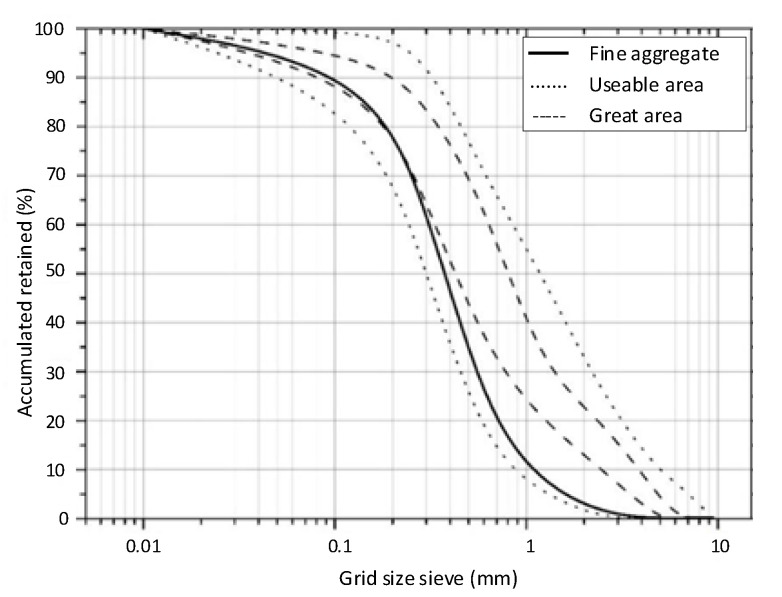
Distribution according to fine aggregate sieve.

The fine aggregate used was quartz sand, whose particles have a fineness modulus of 1.89. The mass specific gravity of the sand is 2.54 g/cm^3^. Figure 1 shows the sieve distribution of fine aggregate.

##### Coarse Aggregate

**Figure 2:**
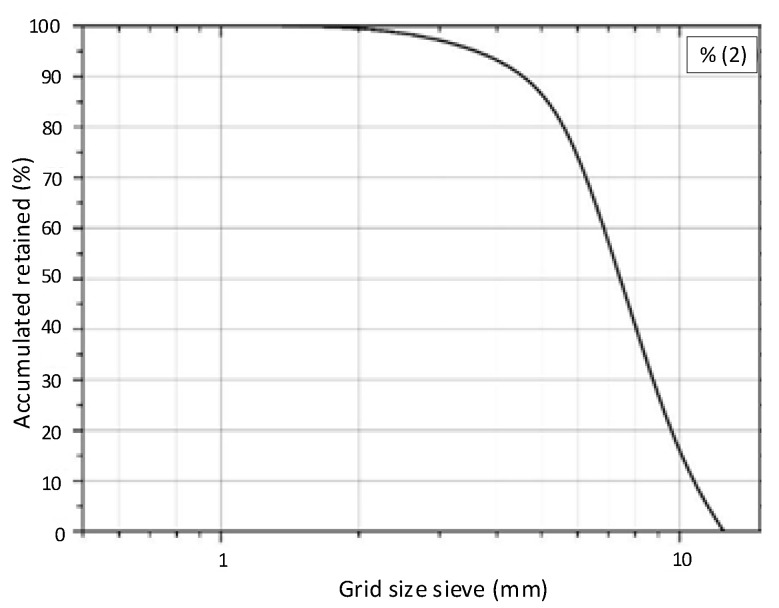
Distribution according to coarse aggregate sieve.

Basalt (Vila Rica, Montenegro, Rio Grande do Sul, Brazil) was used as coarse aggregate, with a mass specific gravity of 3.01 g/cm^3^. Figure 2 shows the sieve distribution of coarse aggregate.

#### 2.1.3. Superplasticizer Additive

The superplasticizer additive (Builder, Cachoeirinha, Rio Grande do Sul, Brazil) was used to maintain the established slump of 220 ± 20 mm, according to NBR NM 67 [33]. This light-yellow liquid is a high-performance superplasticizer additive, with a pH of 4 to 7 and a density of 1.10 ± 0.2 g/mL.

#### 2.1.4. Crystalline Admixtures

Three different powder crystalline admixtures available on the market, called X, Y, and Z, were used. Table 2 presents the laser granulometry, and Table 3 presents the chemical composition of the crystalline admixtures.

#### 2.1.5. Water

The water used in this study for molding the specimens came from the public supply of the city of Porto Alegre, Rio Grande do Sul, Brazil.

### 2.2. Experimental Program

**Figure 3:**
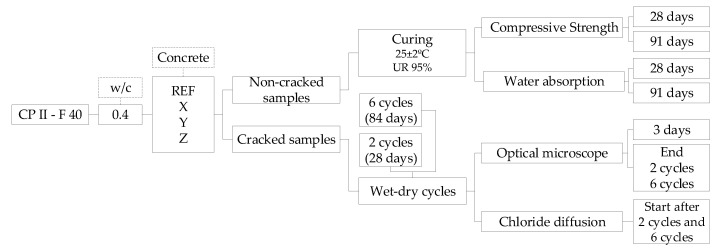
Experimental program.

To achieve the objectives proposed in this research, the experimental program presented in Figure 3 was developed.

#### 2.2.1. Concrete Production

The proportions of each material used are described in Table 4. The w/c (water/cement) ratio was set at 0.4. All concretes were molded following this proportion of materials, varying only the type of crystalline admixtures used at a 1% dosage in the cement mass (this content is recommended by the manufacturers). The molding of the samples was carried out according to NBR 5738 [34]. After mixing the materials, the concrete’s consistency was fixed at 200 ± 20 mm, evaluated through the slump test, as required by NBR NM 67 [33].

Cylindrical specimens with dimensions of 100 mm in diameter by 200 mm in height were molded for the test of resistance to axial compression and water absorption by capillarity, and samples of 95 mm in diameter by 190 mm in height were used for the test optical microscopy and chloride diffusion. The specimens were cut for the chloride diffusion test and optical microscopy, with the5 cm thick upper and lower slices being discarded. For the chloride diffusion test, only central slices of 3 cm thickness each were used.

#### 2.2.2. Crack Creation and Wet–Dry Cycles

For the creation of cracks, the specimens (3 cm thick) were positioned on the press, according to NBR 7222 [35], which establishes the criteria for determining the tensile strength by diametrical compression of cylindrical specimens. Two metal bars with a diameter of 5 mm were used, positioned in the sample’s central region, to direct the crack opening perpendicularly to the load application.

**Figure 4:**
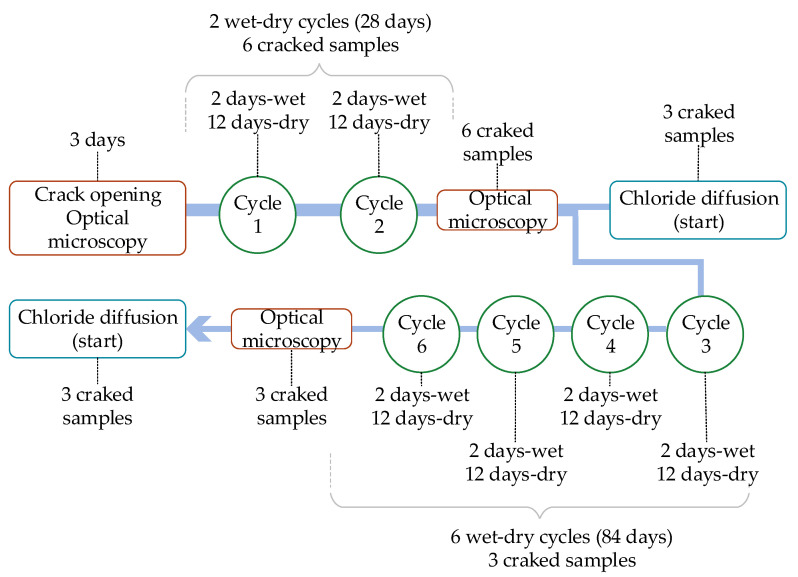
Wet–dry cycles.

After creating the cracks, wetting and drying cycles were performed for 28 days (two cycles) and 84 days (six cycles) in the chloride diffusion test samples; one cycle corresponds to 2 days of wetting and 14 days of drying. The water used for the immersion cycles came from the public supply network, renewed at each new cycle. Figure 4 shows the scheme of the cycles used. The details of the optical microscopy and chloride diffusion assays are specified in the sections below.

#### 2.2.3. Compressive Strength

A simple compressive test, recommended by the NBR 5739 [36], was used to determine resistance at 28 and 91 days.

#### 2.2.4. Capillarity Water Absorption

A capillarity water absorption test was performed according to NBR 9779 [37] at 28 and 91 days.

#### 2.2.5. Crack Width Measurements Using Microscopy

Each specimen’s crack width was measured using an optical microscope (Zeiss Stemi 508, with 2× magnification at 0.65× resolution) (Carl Zeiss Microscopy, New York, NY, USA) on the crack surface. The crack thickness measurement was performed at 3 days, for the selection of cracks with thicknesses less than 0.4 mm, and at the end of the wetting–drying cycles. For each concrete, six cracked specimens were chosen, three for each age of the chloride diffusion test (28 and 84 days). For the images to always be generated in the same place at different ages, 10 zones of 0.6 mm were demarcated in the sample’s central region (95 mm in diameter), discounting the 35 mm of the edges. From each sample for each age, 10 images were always generated in the same place.

Through the images generated, the healing efficiency of the crack could be assessed. The cure rate was calculated using Equation (2).

(2) $$T\;\left(\%\right)=\frac{Nú\mathrm{mC}}{Nú\mathrm{mA}-Nú\mathrm{mB}},$$

where NúmA is the total number of pixels in the image, NúmB is the initial number of white pixels in the sample, and NúmC is the number of black crack pixels.

#### 2.2.6. Chloride Diffusion

**Figure 5:**
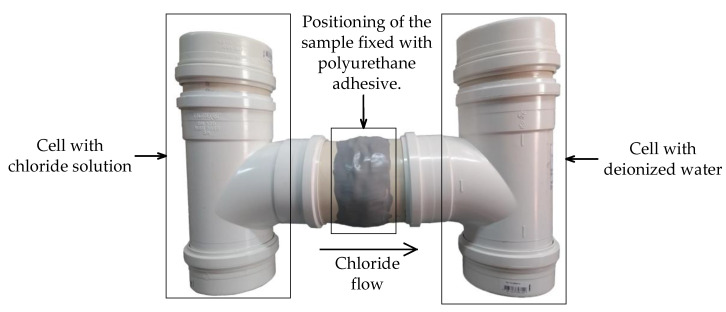
Chloride diffusion testing apparatus.

The chloride diffusion test was used to verify the internal self-healing of cracks, carried out according to UNE 83987 [38]. To simulate a real situation of concrete exposure to a chloride environment, it was decided not to apply the 12 V voltage. For each concrete studied, the test was carried out with three samples of dimensions of 95 mm in diameter and 30 mm in thickness. The apparatus used in the chloride diffusion test was based on the model proposed by UNE 83987 [38], with advantages such as being hermetically sealed to avoid possible chloride contamination by the laboratory and to prevent water evaporation during the test, which ensured the concentration of chlorides in the solution. Figure 5 shows the apparatus for the chloride diffusion test.

The samples were collected in a cell with deionized water to check the chloride passage through the concretes over time. To perform the readings and determine the chloride content, the Hanna Instruments HI 98191 equipment (Hanna Instruments of Brazil, Tamboré, São Paulo, Brazil) and the selective ion electrode HI 4007, from the same manufacturer, were used.

For the calculation of the diffusion coefficient of chlorides and prediction of useful life, Equation (3), from Fick’s second law, was used.

(3) $$\frac{Ccl-Co}{Cs-Co}=1-erf\left(\frac{x}{2\cdot \sqrt{D\cdot t}}\right),$$

where *Ccl* is the chloride concentration at depth *x* and time *t* (mg/L), *Co* is the initial concentration of chlorides within the structural component, *Cs* is the chloride concentration on the surface of the concrete structural component, taken as constant (mg/L), *erf* is the Gauss error function, *x* is the depth considered (cm), *D* is the chloride diffusion coefficient (cm^2^/s), and *t* is the length of time considered (s).

## 3. Results and Discussion

### 3.1. Compressive Strength

**Figure 6:**
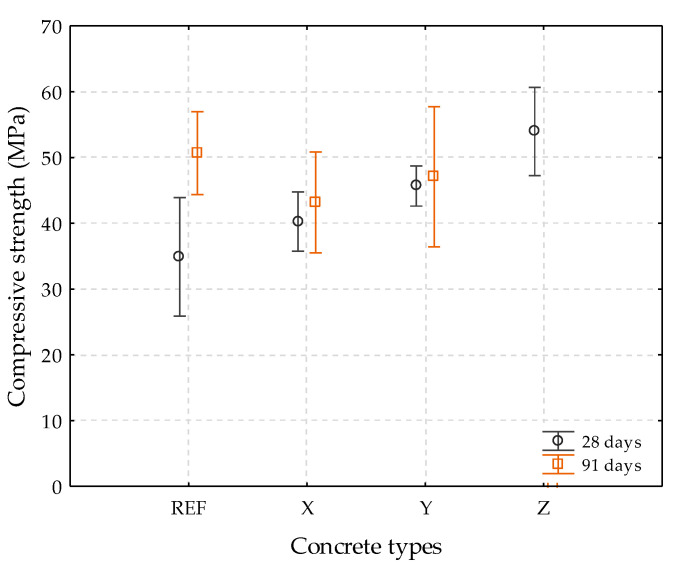
Compressive strength at 28 and 91 days.

The results of the compressive strength test of the specimens are shown in Table 5 and Figure 6. The averages were acquired by breaking four specimens at the age of 28 and 91 days.

By analyzing Table 5 and Figure 6, at 28 days with respect to the reference, concretes containing crystalline admixtures X, Y, and Z showed gains in strength of 15.4%, 30.9%, and 54.6%, respectively. The values obtained for the concrete with crystalline admixture Z were not considered due to the high coefficient of variation presented (13.2%). Roig-Flores et al. [7] reported in their work an increase of 15% in the compressive strength of concretes containing crystalline admixtures at 28 days when compared to a reference concrete. This increase in compressive strength may be related to the filler effect provided by crystalline admixtures, to the point of contributing to the filling of the matrix pores [7]. Furthermore, crystalline admixtures may have acted as an activator of cement hydration and contributed to the densification of C-S-H in the cementitious matrix [36].

Pazderka and Hájková [39] did not obtain a significant gain regarding the compressive strength of concretes containing 2% crystalline admixtures at 28 days. Ferrara and Krelani [40] observed the same behavior, whereby the content of 1% crystalline admixtures did not increase the compressive strength of concrete samples until 30 days when compared to samples without additions.

For the age of 91 days, it can be noted that the concretes containing the crystalline admixtures showed less resistance in the compressive test when compared to the reference. Likewise, Helene et al. [41] demonstrated little contribution of crystalline admixtures to concrete compressive strength, for the same w/c ratios, at the age of 91 days. Munn, Chang, and Kao [42] found that the addition of 0.8% and 1.2% crystalline admixtures did not significantly increase the compressive strength of concretes made with ordinary cement, but found an increase in strength at 28 and 91 days for concretes produced with pozzolanic cement and the addition 1.2% crystalline admixtures. Better results could have been observed if the specimens were exposed to another curing condition, such as complete immersion [26,43].

To study the significance of controllable variables, a variance analysis (ANOVA) was carried out with factors using the software Statistica 13 (StatSoft, Tulsa, OK, USA). The results are shown in Table 6.

It can be seen that the influence of the controllable variables used was considered statistically significant. Thus, the use of crystalline admixtures and the increase in age of the concrete made a difference regarding the results of compressive strength.

### 3.2. Capillarity Water Absorption

**Figure 7:**
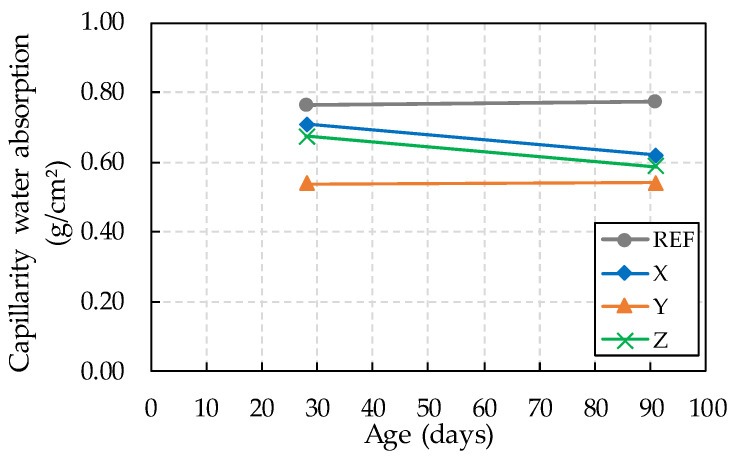
Capillarity water absorption at 28 and 91 days.

The results obtained in the capillarity water absorption test are shown in Table 7 and Figure 7 and correspond to the average of the absorption values after 72 h of testing. The averages were obtained by testing four specimens at 28 and 91 days of age.

The capillarity water absorption mechanism is not only influenced by the characteristics of the liquid, such as density, surface tension, and viscosity, but also depends on the characteristics of the concrete, such as tortuosity, radius, and communication between the capillary pores; the absorption of water is more intense with a smaller diameter of the capillary pores and their connections [23].

Thus, the greater water absorption by capillarity observed in the reference for both ages may be related to the cement used in the present study. CPII-F cement has filler in its composition, a material that is considered to have similar inertness and fineness to cement, acting in the densification of the concrete matrix, causing a reduction in the size of the capillary pores, which in turn increases absorption.

Regarding the effect provided by crystalline admixtures, it is possible to observe a reduction in the absorption of water by capillarity in concretes with these materials. At 28 days compared to the reference, concretes with additives X, Y, and Z showed a reduction of 7%, 41%, and 13%, respectively.

At 91 days, a more considerable effect was observed using these materials, where the reduction in absorption was 24%, 43%, and 31%, respectively, for the crystalline admixtures X, Y, and Z. It is also possible to notice that there was no reduction in absorption from 28 to 91 days in the reference concrete, indicating that the reduction in absorption perceived in other concretes was mainly due to the effect of the crystalline admixtures used.

In the study by Joa et al. [20], 3% crystalline admixtures in mortars provided a reduction in porosity and water absorption. On the other hand, Hassani et al. [16] found no considerable difference in the results of water absorption in concretes containing 1% crystalline admixtures at 28 days.

To study the significance of controllable variables, a variance analysis (ANOVA) was carried out with the software Statistica 13. The results for the capillarity water absorption are shown in Table 8.

### 3.3. Crack Width Measurements Using Microscopy

**Figure 8:**
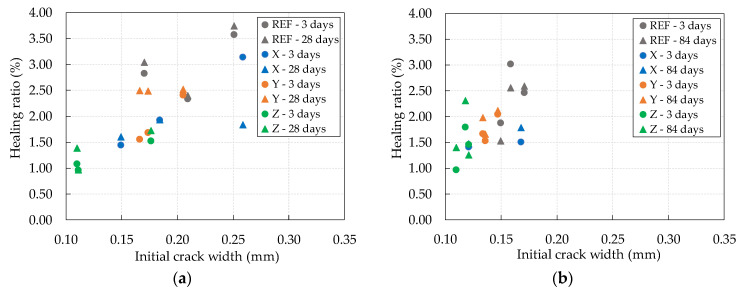
Healing ratio as a percentage versus initial crack width: (**a**) after two wet–dry cycles; (**b**) after six wet–dry cycles.

At 3 days, the crack thickness was measured on its surface, and images of the samples were collected using an optical microscope. Subsequently, after the wet–dry cycles, new images of the samples were collected to assess the crack self-healing rate. The test was performed on the same specimens used for the chloride diffusion test. The evolution of the self-healing of all evaluated samples is shown in Figure 8.

Analyzing the results presented in Figure 8, an increase in the self-healing rate of cracks could be seen for most of the studied concretes in the different wetting/drying cycles applied.

One reason for this increase in the rates of self-healing of cracks was the methodology for quantifying the thicknesses per pixel, using the ImageJ software. As explained in Section 2.2.1 and Section 2.2.2, the specimens were cut to obtain samples with dimensions of 95 mm in diameter and 30 mm in thickness, and then the cracks were created. As can be seen in Figure 8, at 3 days, all samples showed a lower rate of self-healing, being higher after the wetting/drying cycles. It was found that, at 3 days, the crack edge region contained the presence of concrete cutoff remains, which resulted in the initial quantification being superficially lower in the first days.

Over time and with the application of the wetting/drying cycles, these remnants were removed, and an increase in the rate of self-healing was observed, leading to an increase in the crack region. In this way, to be perceived, the self-healing of a crack would need to be superior to the loose fines generated when cutting the specimens. However, this would not be perceived using optical microscopy if the samples were not cut and were molded directly into the dimensions required for testing [24].

With crystalline admixtures, more significant self-healing was expected in most samples, including for greater crack thicknesses. However, a factor considered influential in this low formation of healing products was the lack of moisture, since the concretes were exposed to predominantly dry conditions, where each cycle was formed by only 2 days of wetting and 14 days of drying [7,12,43]. On the other hand, wetting and drying cycles can effectively promote the self-healing of cracks when the sample immersion period is greater than or equal to the applied drying period [12,25,44].

Given the above, it can be seen that, superficially, the self-healing of cracks was not observed. This result is related to the cracking methodology applied, which was not efficient in this work, as well as to the short wetting period that the concretes with crystalline admixtures were exposed to in the wetting/drying cycles.

As already mentioned, superficially, it was impossible to observe any point of self-healing of the cracks in all evaluated concretes. However, through optical microscopy, this behavior could not be confirmed for their internal region. The results showing the internal behavior of self-healing of cracks are presented below.

### 3.4. Chloride Diffusion

**Figure 9:**
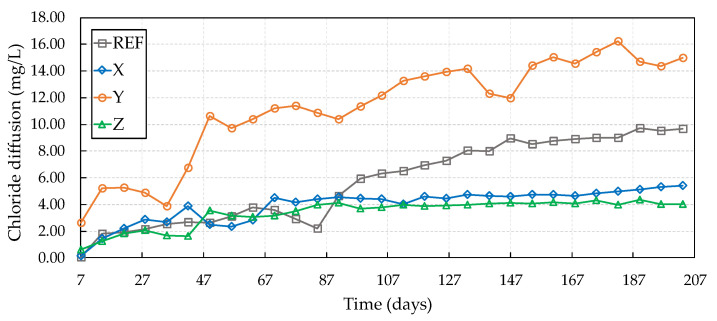
Chloride diffusion after two wet–dry cycles.

The chloride diffusion test was used to check the internal self-healing of cracks. This test started after two and six cycles of wetting and drying, with three cracked samples of each concrete, i.e., the same samples being used in optical microscopy. Figure 9 presents the results obtained for the diffusion of chlorides from the samples submitted to the two wetting and drying cycles (28 days).

It can be observed that, among the crystalline admixtures, X and Z presented the lowest chloride passage over time. The crystalline admixture Y had the worst result during the test, with a more significant passage of chlorides over time, showing itself to be inferior even to the performance of the reference concrete. This result presented by the crystalline admixture Y may be related to its greater granulometry, thereby delaying the hydration reactions of this material before the start of the diffusion test [45]. 

**Figure 10:**
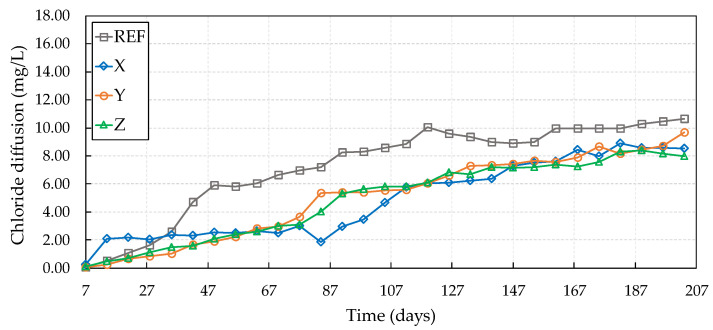
Chloride diffusion after six wet–dry cycles.

Concretes with crystalline admixtures X and Z showed a reduction of 35.55% and 58.53%, respectively, in relation to the reference, while the crystalline admixture Y showed an increase in this passage over time by 52.44%. In this sense, Borg et al. [46] evaluated the self-healing of cracks in concretes with crystalline admixtures as facilitating agents for self-healing when exposed to chloride-rich environments. The authors found that a high hydrophilic reactivity of crystalline admixtures led to the best performance for most of the investigated exposure conditions, even for the sealing of cracks up to 0.3 mm thick. Figure 10 shows the specimen results at the start of the chloride diffusion test after six wetting and drying cycles (84 days).

Analyzing Figure 9 and Figure 10, it can be seen that the cracked samples with crystalline admixtures did not wholly prevent the passage of chlorides through the cracks in the concretes but reduced this passage compared to the reference. In this case, the wetting/drying cycles were not wholly effective in activating and accelerating the crystalline admixture reactions causing the complete self-healing of the cracks before the specimens were subjected to the chloride diffusion test. These results can be related to the predominantly dry cycles applied to the concrete samples (12 days of drying) and to the short wetting period (2 days). It can be noted that, among crystalline admixtures, the most significant reduction in the passage of chlorides through the crack was provided by crystalline admixture Y (66.02%), showing that this additive requires a longer time for hydration reactions due to its greater granulometry.

However, as can be seen, all crystalline admixtures provided a reduction in the passage of chlorides. That is, they acted in reducing the internal thickness of the crack. In this sense, Huang [6] found that the filling of cracks with hydration products was 28% after curing in water for 200 h. This demonstrates that the self-healing of cracks can significantly reduce the entry of aggressive agents through cracks and, thus, prolong the life of concrete structures, even if the cracks are not completely filled with hydration products.

Jacobsen, Marchand, and Boisvert [47] verified the self-healing of cracks in concrete samples after 3 months of immersion in water, observing a reduction in the chloride migration rate. The reduction was 28% to 35% compared to the migration of freshly cracked samples. This effect agrees with the findings of Şahmaran [48], who observed a reduced chloride diffusion coefficient and a slower penetration rate due to the effects of self-healing of cracks.

As previously mentioned, the wetting and drying cycles were not fully effective in causing the cracks to close, mainly due to the short wetting period that the specimens were exposed to. However, after these cycles, the direct contact of concretes containing crystalline admixtures with the water during the diffusion test may have positively influenced the reduction of the passage of chloride ions from the continuous hydration reaction of the crystalline admixtures with the water of the test. This is in line with the results observed by Borg et al. [46], who verified that the ability to seal cracks in concrete continued to occur for samples submerged in water with chlorides. The authors also pointed out that this self-healing may prove to be more efficient than in water without chlorides.

The crack internal morphology is another factor that may have contributed to the greater or lesser passage of chlorides. The cracked specimens used in this work were selected by image analysis; thus, only the crack’s external surface and not its internal configuration was known. According to Roig-Flores et al. [7], a crack’s internal morphology may be responsible for the greater efficiency of self-healing when initiating this phenomenon in internal areas that present lesser thickness of the cracks, even in cases where greater thicknesses are verified on the surface of the samples.

**Figure 11:**
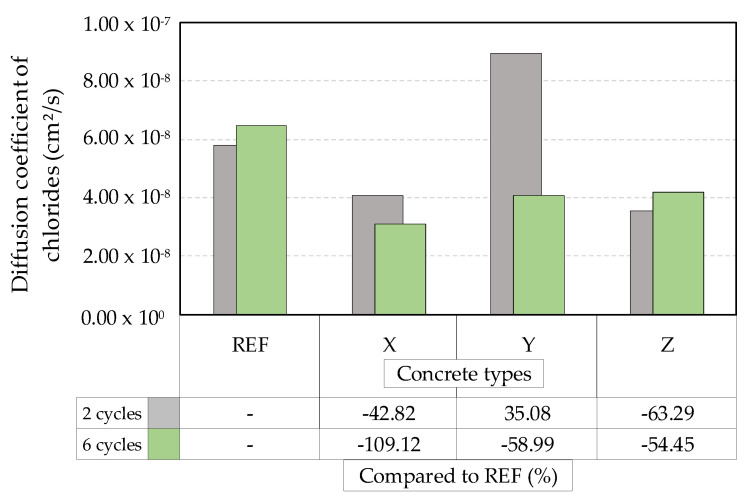
Average diffusion coefficients of concretes after two and six wet–dry cycles.

From the chloride diffusion test and Equation (3) describing Fick’s second law, it was possible to calculate the chloride diffusion coefficient for each studied concrete, a parameter that characterizes the concrete’s resistance to the passage of chlorides. Thus, through Figure 11, it is possible to identify the average results of the chloride diffusion coefficients.

Analyzing Figure 11, it can be seen that, for specimens submitted to two wetting/drying cycles, the diffusion coefficients of concretes with crystalline admixtures X and Z showed a reduction of 42.82% and 63.29%, respectively, with respect to the reference concrete. As previously discussed, concretes with the crystalline admixture Y showed lower resistance to chloride passage during the diffusion test and, thus, obtained a diffusion coefficient of 35.08% higher than the reference. For the six wetting/drying cycles, the three crystalline admixtures with a percentage reduction in relation to the reference stand out, denoting a greater resistance to the entry of chlorides.

Azarsa, Gupta, and Biparva [4] investigated, through different methodologies, the effectiveness of crystalline admixtures in promoting improvements in the self-healing in concretes, in addition to improvements in chloride penetration and water permeability. Regarding the penetration of chlorides, the results indicated that the concrete samples treated with the crystalline admixtures had a lower diffusion coefficient at the two curing ages (28 and 56 days) compared to the control mixture, suggesting the formation of crystalline structures inside the concrete acting as a physical barrier to the passage of chlorides.

Analysis of variance (ANOVA) was performed with the software Statistica 13. The results of the diffusion coefficients are shown in Table 9.

The predicted useful life of concrete structures corresponds to the period that chlorides take to pass through the concrete covering layer until they reach to and dissipate in the reinforcement, which can start corrosion. Therefore, the prediction of useful life is totally influenced by the diffusion coefficient. A higher diffusion coefficient suggests a lower resistance of the concrete to the passage of chlorides and, consequently, a lower calculated useful life.

**Figure 12:**
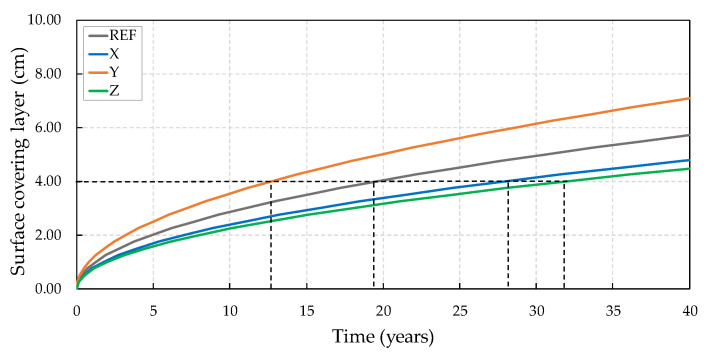
Service life prediction of cracked concretes subjected to two wet–dry cycles.

Thus, through the diffusion coefficients found for the concretes, the useful life was forecast using Equation (3), with the results shown in Figure 12 for concretes subjected to two wetting and drying cycles.

**Figure 13:**
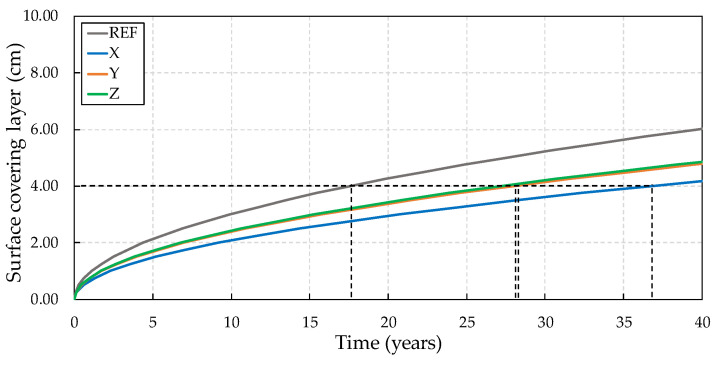
Service life prediction of cracked concretes subjected to six wet–dry cycles.

Considering the 4 cm coverage recommended by the NBR 6118 [49], the reference concrete presented a useful life forecast of 19.6 years until the chlorides would reach the reinforcement. The concrete with crystalline admixture X had a useful life forecast of 28 years, representing an increase of 42.80% with respect to the reference. Among the crystalline admixtures, the worst performance can be noted for Y, with a 13 year life expectancy, showing a 35% reduction in relation to the reference, while crystalline admixture Z showed greater efficiency, with a predicted lifetime of 32 years (an increase of 63.30%). The predicted useful life of concretes subjected to six wetting/drying cycles is shown in Figure 13.

It can be noted that the reference concrete had a useful life forecast of 17.6 years. A better result was noted for concrete with crystalline admixture X (37 years), while concretes with crystalline admixtures Y and Z showed similar performance (approximately 28 years), with a gain of 57% in the time before reaching the reinforcement.

Thus, it can be observed that the use of crystalline admixtures provided a reduction in the passage of chlorides through the concretes during the diffusion test and, consequently, to an extension of the time until these aggressive agents would reach the reinforcement, thereby causing the corrosion to start. It is worth mentioning that the results presented refer to cracked concretes, which allow a more accelerated movement of chlorides through the interior of the cementitious matrix and, as a result, less time for depassivation of the reinforcement.

## 4. Conclusions

This study explored the effects of using crystalline admixtures on the self-healing of cracks in concrete. We reached the following conclusions from the results:

1. From the optical microscopy test, it was observed that the wetting and drying cycles were not efficient in activating the hydration reactions of crystalline admixtures on the sample surface. Thus, they did not provide complete crack sealing due to the short wetting period that the concretes were exposed to.
2. The analysis and quantification of images by optical microscopy was not able to efficiently detect the self-healing of concretes, mainly due to the presence of loose fragments of mortar present on the crack surface due to the cutting procedure of concrete samples.
3. From the chloride diffusion test, it was possible to internally observe the performance of crystalline admixtures and the wetting/drying cycles in the closing of cracks and the reduction of the passage of chlorides in comparison to the reference concrete.
4. The chloride diffusion test proved to be feasible for detecting self-healing in cracked samples. At the different ages of the test (after two and six wetting/drying cycles), it was possible to observe that the crystalline admixtures delayed the passage of chlorides over time with respect to the reference concrete.
5. The concretes with crystalline admixtures showed an increase in the useful life forecast compared to the reference concrete at the two evaluated ages, with the exception of the concrete with crystalline admixture Y when the diffusion test started at 28 days.
6. Regarding the performance of crystalline admixtures, concretes with crystalline admixtures X and Z showed a reduction in chloride passage with respect to the reference concrete, considering the two ages of the chloride diffusion test. Concrete with crystalline admixture Y only showed this reduction after six cycles of wetting and drying.
7. From the results, it was possible to observe that the internal self-healing behavior of a crack can differ from the behavior observed in the outer region. Thus, a precipitous conclusion of self-healing cannot be reached on the basis of the crack’s surface, as the internal behavior can be quite different.

## Figures and Tables

**Table 1:** Physicochemical characterization of CP II-F 40 cement.

Cement CP II-F 40		Content (% by Mass)	
		Real	NL ^1^
Loss to fire		5.20	≤6.5
Silicon dioxide (SiO_2_)		12.78	
Aluminum oxide (Al_2_O_3_)		3.77	
Iron oxide (Fe_2_O_3_)		4.13	
Calcium oxide (CaO)		66.48	
Magnesium oxide (MgO)		1.46	
Sulfur trioxide (SO_3_)		6.84	≤4.0
Sodium oxide (Na_2_O)		-	
Potassium oxide (K_2_O)		1.19	
Carbonic dioxide (CO_2_)		2.93	≤5.0
Laser granulometry (µm)	Average diameter	12.84	
	Diameter 10%	0.34	
	Diameter 50%	2.13	
	Diameter 90%	35.78	
Mass specific gravity (g/cm^3^)		3.11	
Specific area (m^2^/g)		5.79	
Compressive strength (MPa)	Day 3	30.28	≥15.0
	Day 7	37.58	≥25.0
	Day 28	42.88	≥40.0

NL^1^: normative limits, according to NBR 11578 [27].

**Table 2:** Laser granulometry of crystalline admixtures.

Granulometry (µm)	Crystalline Admixture X	Crystalline Admixture Y	Crystalline Admixture Z
Diameter at 10%	0.92	1.73	1.00
Diameter at 50%	8.14	11.63	9.06
Diameter at 90%	22.96	30.74	23.11
Average diameter	10.11	14.42	10.72

**Table 3:** Chemical composition of crystalline admixtures.

Content (% by Mass)			
Chemical Composition	Crystalline Admixture X	Crystalline Admixture Y	Crystalline Admixture Z
Loss to fire	17.81	12.87	8.57
Silicon dioxide (SiO_2_)	8.94	12.64	13.07
Aluminum oxide (Al_2_O_3_)	2.67	2.95	3.80
Iron oxide (Fe_2_O_3_)	2.24	2.50	2.81
Calcium oxide (CaO)	64.07	58.45	60.61
Magnesium oxide (MgO)	0.55	5.54	4.81
Sulfur trioxide (SO_3_)	2.44	3.43	4.43
Sodium oxide (Na_2_O)	0.00	0.00	0.00
Potassium oxide (K_2_O)	0.56	1.04	1.22
Chlorine (Cl)	0.15	0.09	0.11
Titanium dioxide (TiO_2_)	0.19	0.18	0.24
Vanadic anhydride (V_2_O_5_)	0.01	0.01	0.02
Manganese oxide (MnO)	0.06	0.06	0.07
Zinc oxide (ZnO)	0.02	N.D.	0.05
Strontium oxide (SrO)	0.28	0.24	0.14
Zirconium dioxide (ZrO_2_)	N.D.	N.D.	0.01
Barium oxide (BaO)	N.D.	N.D.	0.02

**Table 4:** Mix proportions in kg.

Concrete	Cement	Fine Aggregate	Coarse Aggregate	w/c (Water/Cement)	Crystalline Admixtures (%) ^1^	Superplasticizer Additive (%) ^1^	Cement Consumption (kg/m^3^)	Scattering (mm)
Reference concrete	1	1.37	2.18	0.40	–	0.09	503.74	200
X					0.10	0.13		200
Y					0.10	0.16		200
Z					0.10	0.14		210

^1^ With respect to the cement mass.

**Table 5:** Compressive strength at 28 and 91 days.

Concrete	28 Days			91 Days		
	Average Strength (MPa)	SD ^1^ (MPa)	CV ^1^ (%)	Average Strength (MPa)	SD (MPa)	CV (%)
Reference	34.9	3.6	10.4	50.7	2.5	5.0
X	40.3	1.8	4.5	43.2	3.1	7.1
Y	45.7	1.2	2.7	47.1	4.3	9.1
Z	54.0	2.7	5.0	-	-	-

SD ^1^, standard deviation; CV ^1^, coefficient of variation.

**Table 6:** Analysis of variance (ANOVA) of the compressive strength.

Effects	QS ^1^	DF ^1^	MS ^1^	Test F	*p* ^1^	Sig.
Concrete	1317.75	3	439.25	58.928	0.000000	Yes
Age	430.12	1	430.12	57.704	0.000001	Yes
Concrete × Age	4325.21	3	1441.74	193.419	0.000000	Yes
Error	119.26	16	7.45	-	-	-

QS ^1^, quadratic sum; DF ^1^, degrees of freedom (n − 1); MS ^1^, mean square; *p* ^1^ < 0.05 denotes a significant effect.

**Table 7:** Capillarity water absorption at 28 and 91 days.

Concrete	28 days			91 days		
	Average Absorption 72 h (g/cm^2^)	SD ^1^ (g/cm^2^)	CV ^1^ (%)	Average Absorption 72 h (g/cm^2^)	SD ^1^ (g/cm^2^)	CV ^1^ (%)
Reference	0.76	0.05	6.80	0.77	0.05	6.69
X	0.71	0.10	14.10	0.62	0.07	11.52
Y	0.54	0.03	6.15	0.54	0.04	6.79
Z	0.67	0.10	14.64	0.59	0.03	5.40

SD ^1^, standard deviation; CV ^1^ coefficient of variation.

**Table 8:** Analysis of variance (ANOVA) of the capillarity water absorption.

Effects	QS ^1^	DF ^1^	MS ^1^	Test F	*p* ^1^	Sig.^1^
Concrete	0.213	3	0.071	16.874	0.000004	Yes
Age	0.013	1	0.013	3.117	0.090225	No
Concrete × Age	0.017	3	0.006	1.323	0.289969	No
Error	0.101	24	0.004	-	-	-

QS ^1^, quadratic sum; DF ^1^, degrees of freedom (n − 1); MS ^1^, mean square; *p* ^1^ < 0.05 denotes a significant effect.

**Table 9:** Analysis of variance (ANOVA) of the diffusion coefficients.

Effects	QS ^1^	DF ^1^	MS ^1^	Test F	*p* ^1^	Sig.
Concrete	4.10 × 10^−15^	3	1.37 × 10^−15^	20.789	0.000009	Yes
Age	7.92 × 10^−16^	1	7.92 × 10^−16^	12.053	0.003146	Yes
Concrete × Age	3.07 × 10^−15^	3	1.02 × 10^−15^	15.552	0.000053	Yes
Error	1.05 × 10^−15^	16	6.57 × 10^−17^	-	-	-

QS ^1^, quadratic sum; DF ^1^, degrees of freedom (n − 1); MS ^1^, mean square; *p* ^1^ < 0.05 denotes a significant effect.
